# Computerised adaptive testing across the paranoia continuum

**DOI:** 10.1136/bmjment-2025-302099

**Published:** 2025-11-12

**Authors:** Daniel Freeman, Sinéad Lambe, Felicity Waite, Laina Rosebrock, Anthony Morrison, Kate Chapman, Robert Dudley, Stephanie Common, Julia Jones, Thomas Kabir, Ariane Beckley, Verity Westgate, Natalie Rouse, Bao Sheng Loe

**Affiliations:** 1 Experimental Psychology, University of Oxford, Oxford, England, UK; 2 Oxford Health NHS Foundation Trust, Oxford, England, UK; 3 Greater Manchester Mental Health NHS Foundation Trust, Manchester, UK; 4 Avon and Wiltshire Mental Health Partnership NHS Trust, Bath, UK; 5 Cumbria Northumberland Tyne and Wear NHS Foundation Trust, Newcastle upon Tyne, England, UK; 6 University of York, York, UK; 7 Tees, Tees Esk and Wear Valleys NHS Foundation Trust, Darlington, England, UK; 8 Department of Psychiatry, University of Oxford, Oxford, UK; 9 The Psychometrics Centre, University of Cambridge, Cambridge, UK

**Keywords:** Psychotic Disorders, Paranoid Disorders, Schizophrenia, Psychological Tests

## Abstract

**Background:**

To drive improvement in clinical services, an important innovation will be to regularly assess patients’ psychotic experiences in order to guide, monitor and, when needed, alter treatment provision. The great heterogeneity in presentations of psychosis means that a comprehensive assessment battery is impractical. A plausible solution is computerised adaptive testing (CAT), which uses real-time computation to present the most informative questions to an individual. Fewer questions are needed to reach similar precision as a full questionnaire.

**Objective:**

We tested the potential of a CAT for paranoia to halve the number of items that need to be presented.

**Methods:**

We used the established item response theory psychometric properties of the 10-item Revised Green *et al* Paranoid Thoughts Scale (Persecution) to run CAT simulations in four datasets in which participants had completed the full scale: a representative survey of 10 382 UK adults; a clinical trial with 319 patients with psychosis; a cohort study of 836 National Health Service (NHS) male patients with psychosis; and a clinical trial with 89 patients with persecutory delusions. The CAT algorithm used the graded response model and the test was concluded when the SE of estimation dropped below 0.3 or five items had been answered.

**Findings:**

On average, the CAT administered 4.2, 4.0, 4.2 and 4.0 items to each person in the four datasets. The correlations between the CAT score and the full-scale paranoia score were 0.95, 0.94, 0.94 and 0.87. Minimal systematic error in paranoia estimation occurred (mean bias scores=−0.01, –0.06, −0.07 to –0.10). Estimation was the least precise for people at the boundary of normal and elevated levels of paranoia.

**Conclusions:**

In datasets with people across the whole paranoia continuum, accurate estimates of paranoia can be provided by a CAT with fewer than half the items of the full scale. Tailored testing may work well with people with psychosis.

**Clinical implications:**

CAT may be a way to implement informative measurement-based care in psychosis services.

**WHAT IS ALREADY KNOWN ON THIS TOPIC**

- Computerised adaptive testing (CAT) (‘tailored assessment’)—presentation of the most informative questions to a person based on their previous answers—quickens assessment. It has rarely been used with patients with psychosis and never using fully continuum-based individual psychotic experience assessments.

**WHAT THIS STUDY ADDS**

- In simulations using datasets with adults from the general population, patients with psychosis in clinical services and patients with persecutory delusions in clinical services, the results show consistently that a CAT for paranoia can efficiently, accurately and precisely estimate a person’s level of paranoia.

**HOW THIS STUDY MIGHT AFFECT RESEARCH, PRACTICE OR POLICY**

- Repeated assessment of psychotic experiences and other important outcomes is a means to improve outcomes for individual patients and the performance of clinical services. CAT could provide a means to implement successfully such change in practice in psychosis services.

## Introduction

 Accurate, patient-centred assessment can drive change in outcomes for individual patients and services. Ideally, a patient entering clinical services for the treatment of psychosis would be formally assessed for the severity of each psychotic experience. This assessment would be repeated weekly or fortnightly after treatment starts. The information gathered would be regularly reviewed in order to monitor the success of an intervention and inform judgements about possible changes to provision. Such information could also be analysed at the aggregate service level to see how outcomes compare both to benchmarks and provision in similar psychosis services. Gathering such patient-reported data at scale could be used to identify which interventions work best for typical presentations and help the personalisation of care. The nearest realisation of such measurement-based care in the UK is National Health Service (NHS) services for the treatment of anxiety and depression. Here, outcome data are collected for almost every treatment session and anonymised data are made publicly available.^1^ It is well-recognised that measurement can also improve psychosis services.^2 3^ Setting standards for treatment provision and regular audits has substantially improved early intervention for psychosis services in the UK.^4^ Measurement-based care is perceived positively by patients with psychosis,^5^ especially when used in shared decision-making. However, there is a particular measurement challenge for psychosis. It is difficult to assess the wide variety of psychotic experiences in a way that is practical to implement, relevant to patients and clinicians and sufficiently accurate to be sensitive to change. In this paper, we examine a way to tailor the assessment of psychotic experiences. This method may enable quicker testing of each psychotic experience type and therefore facilitate comprehensive coverage of many psychotic experiences.

Psychotic experiences are, of course, what leads a person to enter psychosis services. The main treatments are designed to reduce these experiences, while other interventions may address the consequences of these difficulties. Patient-reported outcomes should be prioritised since, whatever inaccuracies self-report can sometimes bring, the patient is still best placed to judge their own experience and they are the recipient of treatment. A key challenge for assessment stems from the complexity of psychosis. Factor analysis indicates at least half a dozen independent dimensions of psychotic experiences^6^,^9^: paranoia, grandiosity, hallucinations, cognitive disorganisation, anhedonia and blunted affect. Patients can also have substantial difficulties with anxiety, depression, sleep, cognitive functioning and social and occupational functioning. Rightly, services would not want patients to complete multiple assessments, of which a substantial portion would not be relevant for an individual.

The value of outcome assessments depends on high levels of completion throughout treatment. This will only occur if patients see the relevance of the questions asked. In recognition of the importance of routine outcome measurement in clinical services, consensus patient-reported outcome measure batteries have been recommended. However, there is a trap in such processes: an understandable wish to reduce assessment length means a focus only on areas that are relevant to all patients. The variability in presentations is not captured. This means assessment of psychotic experiences can fall by the wayside. Since there is such a wide variety of psychotic experiences and not all patients will experience any one symptom, batteries can end up focusing on generic concerns and do not actually assess psychotic experiences.^2^ When psychotic experiences have been included, there have been only a very few questions in a lengthy battery covering many different other outcome areas.^10^

These consensus batteries have relied on assessments based on traditional standardised testing methods (classical test theory), which provide a fixed set of items to every test taker. Typically, each item is weighted equally to produce a total score for each clinical area. Modern test theory—item response theory (IRT)—provides an alternative approach.^11^ Here, the focus is on individual items, with probabilistic models used to determine the properties of an item in relation to the construct (the trait) being measured. IRT provides information on item location (where it best functions across the severity of the trait being measured), discrimination (how well it differentiates between people at different trait levels) and position on the trait scale.^12^ In computerised adaptive testing (CAT),^13^,^15^ this information about each item in a scale is used to tailor the assessment for each individual. Each response is dynamically used to provide an estimate of the construct score that is then used to select the next item (maximum information item selection rule). This focus on presenting the most informative item for a person at any one time improves the efficiency of test administration, as not all test items will need to be answered. In a CAT, the number of items presented will vary across individuals, and different stopping rules can be applied—for example, the measurement error of the final score or the number of items to be presented. Typically, more items need to be presented at a boundary in a trait—either a low or high score. But it is generally considered that less precision is needed at these points since either a person does not have a significant difficulty with the trait or it is clear that they are among the most affected.^13^

CAT has been applied to psychosis. For example, CAT-Psychosis used an item pool from standard clinical psychiatric assessments such as the Scale for the Assessment of Positive Symptoms.^16^ The CAT was developed and tested with 649 patients with psychosis. An average presentation of 12 items provided an accurate estimate of total symptom score in the patient group.^17^ Our view is that there are multiple independent spectra of psychotic experiences across the general population, that is, that they are quantitative traits. Each has variation in causes.^18^ Each may be the focus of treatment, each may need different treatment approaches and each may lead to different trajectories of outcomes. Hence, separate fully dimensional assessments are required. Therefore, a CAT is needed that provides dimensional symptom scores, usable in clinical and non-clinical populations, across multiple different psychotic experiences (eg, paranoia, grandiosity, hallucinations, anhedonia).

## Objective

The first step was to assess whether a CAT approach can be successfully applied across the full spectrum of severity of a single psychotic experience. The focus was on paranoia. Paranoia is the inaccurate idea that others intend harm to the individual.^19^ About one in five of the population has regular paranoid thoughts.^20^ Paranoia can occur at higher rates in many psychiatric conditions.^21 22^ At the severe end of the paranoia continuum are persecutory delusions. Persecutory delusions are one of the most common difficulties for people diagnosed with psychosis.^23 24^ We have previously shown in a CAT simulation of an adolescent paranoia assessment that the number of items could be reduced.^25^ Here, we evaluated the potential of a CAT for the leading dimensional paranoia assessment, the 10-item Revised Green *et al* Paranoid Thoughts Scale (R-GPTS) Part B (persecutory ideation).^26^ This assessment is commonly used in research and treatment trials.^27^,^29^ The R-GPTS was developed with over 2000 patients with psychosis and over 10 000 non-clinical individuals. IRT was used in the evaluation of the R-GPTS, meaning that a calibrated item bank was available to use in a CAT. We set out to simulate the use of the CAT in *new* datasets from non-clinical and clinical populations. Our objective was to evaluate whether it is possible with a CAT to halve the number of items administered but maintain accuracy in the assessment of paranoia.

## Methods

### Participants

Four datasets were used in which the full paranoia measure had been administered. First, data collected in March 2023 from 10 382 UK adults, quota sampled to match the UK population for age, gender, ethnicity, income and region.^20^ Second, baseline data collected from 319 adult patients with psychosis attending NHS mental health trusts who were taking part in the gameChange clinical trial.^30^ These data were collected from July 2019 to May 2021. Third, data collected from February 2023 to March 2024 from 836 adult male patients with psychosis attending NHS mental health trusts.^31^ Fourth, baseline data collected from 89 patients with current persecutory delusions (in the context of psychosis) attending NHS mental health trusts who were taking part in the evaluation of Feeling Safer (a 6-month supported online programme). We included baseline data from the first 14 patients in the initial proof-of-concept testing of Feeling Safer collected from April 2024 to August 2024^32^ and the first 75 patients randomised into the full clinical trial from January 2025 to May 2025.^27^

### Assessment

*R-GPTS part B (persecutory ideation*):^26^ the scale items were generated based on a definition that persecutory ideation consists of believing that harm is going to occur and that the perpetrator has the deliberate intention to cause this harm.^19^ The scale comprises 10 items (eg, ‘Certain individuals have had it in for me’, ‘I was convinced there was a conspiracy against me’, ‘People have been hostile to me on purpose’) each rated for the past month on a five-point scale from 0 (not at all) to 4 (totally). The items form a single factor.^20 26^ A differential item functioning (DIF) analysis for age and gender showed that all items were invariant between men and women and between age groups.^26^ Measurement invariance for the Part B scale has also been shown across several countries.^33^ Performance across ethnicity for the R-GPTS Part B had not been examined. Therefore, we also conducted new DIF analyses with three large datasets that showed invariance between white and all other ethnic groups (see online supplemental materials). Higher scores on the R-GPTS indicate higher levels of paranoia. Questionnaire summary scores across the participant groups are shown in table 1. The IRT psychometric properties are summarised in table 2.

**Table 1:** R-GPTS part B (persecution) scores in the four groups

Study	N	Mean	SD	Lowest score	Maximum score
UK adult representative population	10 382	9.7	11.5	0	40
gameChange patients with psychosis	316	15.7	12.9	0	40
Adult male patients with psychosis	828	12.4	12.9	0	40
Feeling Safer patients with persecutory delusions	88	29.5	9.1	5	40

R-GPTS, Revised Green et al Paranoid Thoughts Scale.

**Table 2:** Psychometric properties of the R-GPTS part B (persecution) from Freeman *et al*^26^

Item	R-GPTS (persecution)	a	b1	b2	b3	b4
1	Certain individuals have had it in for me	2.95 (0.06)	0.20 (0.01)	0.71 (0.02)	1.28 (0.02)	1.70 (0.03)
2	People wanted me to feel threatened, so they stared at me.	2.87 (0.06)	0.75 (0.02)	1.14 (0.02)	1.58 (0.02)	2.02 (0.03)
3	I was certain people did things in order to annoy me	2.43 (0.04)	−0.10 (0.02)	0.46 (0.02)	1.08 (0.02)	1.61 (0.03)
4	I was convinced there was a conspiracy against me.	3.68 (0.08)	0.79 (0.02)	1.08 (0.02)	1.43 (0.02)	1.75 (0.03)
5	I was sure someone wanted to hurt me	4.25 (0.10)	0.78 (0.01)	1.08 (0.02)	1.39 (0.02)	1.68 (0.03)
6	I couldn't stop thinking about people wanting to confuse me	3.15 (0.07)	0.65 (0.02)	1.02 (0.02)	1.49 (0.02)	1.89 (0.03)
7	I was distressed by being persecuted	4.45 (0.10)	0.77 (0.01)	1.07 (0.02)	1.40 (0.02)	1.75 (0.02)
8	It was difficult to stop thinking about people wanting to make me feel bad	3.99 (0.08)	0.40 (0.01)	0.79 (0.02)	1.21 (0.02)	1.59 (0.02)
9	People have been hostile towards me on purpose.	3.54 (0.07)	0.35 (0.01)	0.79 (0.02)	1.24 (0.02)	1.63 (0.02)
10	I was angry that someone wanted to hurt me.	3.59 (0.08)	0.63 (0.01)	0.98 (0.02)	1.35 (0.02)	1.69 (0.02)

a=discrimination, b=difficulty parameters at the category thresholds between 0–1 (b1), 1–2 (b2), 2–3 (b3) and 3–4 (b4).

R-GPTS, Revised Green *et al* Paranoid Thoughts Scale.

### Analysis

The CAT algorithm was developed on the basis of the item parameters from the IRT analyses conducted when developing the R-GPTS; that is, the scale items were precalibrated. The four datasets for the CAT simulations were independent of the original R-GPTS calibration dataset. By feeding real response data into a CAT simulation, a wide array of performance metrics can be assessed, including efficiency (how many items are needed), accuracy (mean bias relative to full-test scores), item exposure rates (the proportion of test administrations in which an item is used) and measurement precision (average SE) of the CAT algorithm. Mean bias is a measure of systematic error, for example, whether on average the measurement method (ie, CAT) overestimates or underestimates scores compared with a reference. In the CAT simulations under discussion, two types of theta values are distinguished: true theta and estimated theta.^34^ True theta refers to the paranoia estimate derived from participants’ responses to all items in the full-length test (ie, the complete R-GPTS Part B) and serves as a proxy for the underlying latent trait. Estimated theta refers to the paranoia estimate generated from a CAT simulation, in which a participant answers only a subset of items selected adaptively. Theta refers to an individual’s estimated ability, often standardised across the group with a mean of 0 and a SD of 1.

We conducted a simulation for each dataset to evaluate the CAT methodology. The algorithm was implemented using the graded response model as the underlying IRT model, which is consistent with the IRT model used to calibrate the items.^35^ The item selection during the adaptive procedure was based on maximum likelihood estimation, and we employed a randomesque method in which the following item was randomly selected from among the top five most informative items.^36^ A dual stopping rule was used to terminate the test: measurement precision and test length. Specifically, the test concluded when the SE of estimation dropped below 0.3 or when the participant had responded to at most five items, whichever criterion was met first. A SE threshold of 0.3 is widely regarded as an appropriate and evidence-based stopping rule in CAT. This threshold corresponds to a reliability of approximately 0.91, which is considered acceptable for diagnostic feedback. Furthermore, we chose to terminate at a maximum of five items because we wanted to test the accuracy of shortening the test significantly. The final paranoia level (theta) estimate for each person was derived using Expected A Posteriori estimation, one of the most widely used approaches due to its ability to produce more stable estimates when test lengths are short or when scores are produced at extreme values (ie, all items are correct or incorrect).^12^ The overall theta estimates for all participants were divided into deciles to evaluate the performance of the CAT across different levels of the paranoia continuum. This enabled an assessment of measurement precision and potential bias at various levels of paranoia severity.

We evaluated the CAT approach not only for its efficiency but also for its psychometric accuracy, particularly the agreement between CAT-based theta estimates and full-test theta scores. Since we had access to complete response data from participants who answered all 10 items of the R-GPTS, we were able to calculate the full-test theta scores to represent the ‘true’ underlying trait levels. We then simulated CAT administration using the same item pool and compared the estimated thetas from the adaptive approach to that of the full-test theta scores. Consistency was evaluated primarily through the correlation between the CAT-derived theta estimates and the full-length theta scores. A Bland-Altman plot was also used to evaluate agreement between estimated thetas and full-test theta values.^37^ This analysis considers the systematic bias and variability of the estimation procedure across the paranoia continuum and provides a visual representation of the extent and pattern of disagreement between the two measurement methods. It is based on the limits of agreement (typically ±1.96 SD of the differences), which encompass approximately 95% of the observed differences under the assumption of normality, enabling assessment of the degree to which a CAT version of the test could reproduce comparable results to the full assessment.

We also examined a specific boundary point for potential classification: when paranoia levels are within the normal range but close to elevated (ie, are approaching potentially problematic levels of paranoia). The R-GPTS development paper set out five ranges of paranoia: average (score 0–5); elevated (0.40–0.75 SD above the average score) (score 6–10); moderately severe (0.80–1.05 SDs above the average score) (score 11–17); severe (1.10–1.45 SDs above the average score) (score 18–27) and very severe (1.50+SDs above the average score) (score 28 or higher). In the general population sample in the development paper, the proportions in each range were: 73% (average), 12% (elevated), 8% (moderately severe), 6% (severe) and 1% (very severe). Based on the elevated level of paranoia, ranging from 0.40 to 0.75 SD above the average, we aimed to evaluate the accuracy of the estimated theta, particularly with early stopping decisions. If an individual’s theta is highly likely to fall below the lower bound of this elevated range (< 0.40), test administration may be justifiably terminated early, as precise estimation is less critical in this context. Nevertheless, examining the accuracy of estimated theta scores in this lower range remains important to ensure reliable classification. Thus, we amended the sample to include only individuals with true theta values below 0.40. We subsequently examined the root mean square error (RMSE) and mean bias of their estimated theta scores to assess how accurately the CAT performed for individuals at this lower end of the paranoia continuum. We repeated the simulation 50 times and calculated the average RMSE and mean bias across runs to ensure the stability and robustness of these estimates. The RMSE provides a summary of the expected deviation between the estimated theta values and their true values. Based on van der Linden and Glas,^38^ RMSE <0.3 is typically regarded as high precision, 0.30 to 0.50 reflects acceptable precision and >0.5 indicates moderate to low precision.

## Findings

### UK adult representative dataset (n=10 392)

On average, the CAT administered 4.24 items per simulee. There was a very high correlation of r=0.947 between the true and estimated paranoia scores, with a RMSE of 0.3301 and a near-zero mean bias of −0.012, indicating both accuracy and minimal systematic error in paranoia estimation. Given that the first item is always the same, the item exposure analysis revealed that only the first item reached the maximum exposure of 1, while the lowest exposure was 0.20. The overall item overlap rate across participants was 0.526, reflecting moderate test content sharing. Table 3 presents conditional CAT performance metrics across the deciles of paranoia in the participant group (D1–D10).

**Table 3:** CAT simulation results: adult representative population and gameChange patients

Deciles	Mean trait	RMSE	Mean bias	Mean test length	Mean SE	Number of simulees
UK adult representative population (n=10 382)						
D1	−0.961	0.285	−0.058	5.000	0.632	1040
D2	−0.915	0.320	−0.037	5.000	0.642	1047
D3	−0.849	0.325	0.013	5.000	0.643	1036
D4	−0.755	0.383	0.055	5.000	0.637	1030
D5	0.157	0.493	0.106	5.000	0.393	1039
D6	0.307	0.345	−0.075	4.203	0.306	1037
D7	0.683	0.298	0.093	3.169	0.279	1038
D8	0.995	0.252	0.072	3.025	0.267	1039
D9	1.272	0.234	0.016	3.064	0.266	1037
D10	1.769	0.280	0.062	4.059	0.294	1039
gameChange patients with psychosis (n=319)						
D1	0.949	0.430	0.174	5.000	0.634	32
D2	0.815	0.465	0.160	5.000	0.640	32
D3	0.075	0.435	0.009	4.938	0.383	32
D4	0.357	0.342	−0.193	4.156	0.302	32
D5	0.619	0.294	0.122	3.281	0.278	32
D6	0.877	0.271	−0.081	3.000	0.266	31
D7	1.119	0.262	0.048	3.031	0.263	32
D8	1.333	0.232	0.079	3.125	0.269	32
D9	1.635	0.245	0.075	3.938	0.266	32
D10	2.157	0.367	−0.017	4.969	0.347	32

D1=lowest 10% (least paranoia), D10=highest 10% (most paranoia). Mean trait=true theta (standardised score) ie paranoia level. RMSE values < 0.3 = high precision and 0.30 to 0.50 = acceptable precision. Mean bias reflects accuracy (negative scores=underestimate, positive scores=overestimate). Mean SE reflects measurement precision (lower=more precise; threshold <0.3).

CAT, computerised adaptive testing; RMSE, root mean square error.

The Bland-Altman plot in online supplemental material figure S1 shows a slight negative bias (−0.012), with a mean difference just below zero, indicating that the simulation tended to, on average, slightly underestimate paranoia levels. The majority of points fall within the 95% limits of agreement, which range approximately from −1.0 to +0.5, suggesting that the estimation error was generally within acceptable bounds. A small number of respondents (n=727; 7.0%) fell outside these limits, particularly at the lower end of the paranoia continuum, where estimation appears less precise. Overall, the plot suggests that the simulation provides reasonably accurate theta estimates across most of the paranoia continuum, with only mild systematic bias and a small proportion of outliers.

Based on 50 simulation runs, the subset of individuals with true theta values below 0.4 (ie, in the normal range for paranoia scores) yielded an RMSE of 0.357 and a mean bias of +0.051 based on the estimated theta values. The RMSE falls within the acceptable precision level. The mean bias reflects a slight systematic overestimation, but its magnitude remains small and can be regarded as negligible. We examined the impact of using estimated theta values and found that approximately 4.1% (n=426) of participants with true theta below 0.4 had an estimated theta that mistakenly placed them in the elevated thresholds, suggesting a low false positive rate. The mean true theta among the misclassified individuals (ie, true value <0.4 but estimated value ≥0.4) was 0.204, corresponding to an expected test score of 4.30.

### GgameChange trial patients with psychosis (N=319)

On average, the CAT administered 4.02 items per simulee. There was a high correlation, r=0.942, between the true and estimated paranoia scores, with a RMSE of 0.342 and a low mean bias of −0.06, suggesting on average a slight underestimation of paranoia. The item exposure analysis showed that only the first item reached the maximum exposure of 1, while the lowest exposure was 0.254. The overall item overlap rate across simulees was 0.51, reflecting moderate test content sharing. Table 3 presents conditional CAT performance metrics across the deciles of paranoia in the participant group.

The Bland-Altman plot in online supplementalmaterial figure S2 shows a slight negative bias (−0.06), with a mean difference just below zero, indicating that the simulation tended to, on average, slightly underestimate paranoia levels. The majority of points fall within the 95% limits of agreement, which range approximately from −1.0 to +0.5, suggesting that the estimation error was generally within acceptable bounds. A small number of respondents (n=15) (4.7%) fell outside these limits, particularly at the lower end of the paranoia continuum, where estimation appears less precise. Overall, the plot suggests that the simulation provides a reasonably accurate theta estimate across most of the paranoia continuum, with only mild systematic bias and a small proportion of outliers.

Based on 50 simulation runs, the subset of individuals with true theta values below 0.4 yielded a mean RMSE of 0.395 and a mean bias of −0.037 based on the estimated theta values. The RMSE falls within the acceptable precision level. The mean bias score reflects a negligible systematic underestimation. Examining the impact of using estimated theta values showed that 6.4% (n=20) of participants with true theta below 0.4 had an estimated theta that mistakenly placed them in the elevated thresholds, suggesting a low false positive rate. The mean true theta among the misclassified individuals was 0.213, corresponding to an expected test score of 4.312.

### Male patients with psychosis attending NHS mental health trusts (n=836)

On average, the CAT administered 4.228 items per simulee. There was a high correlation, r=0.94, between the true and estimated paranoia values, with a RMSE of 0.372 and a mean bias of −0.066, suggesting on average a slight underestimation of paranoia. Only the first item reached the maximum exposure of 1, while the lowest exposure was 0.218. The overall item overlap rate across simulees was 0.5213, reflecting moderate test content sharing. Table 4 presents conditional CAT performance metrics across the deciles of paranoia in this group (D1–D10).

**Table 4:** CAT simulation results: adult male patients with psychosis and Feeling Safer patients

Deciles	Mean trait	RMSE	Mean bias	Mean test length	Mean SE	Number of simulees
**Adult male patients with psychosis (n=836)**						
D1	−0.948	0.426	−0.162	5.000	0.634	85
D2	−0.875	0.461	−0.155	5.000	0.643	83
D3	0.748	0.370	0.030	5.000	0.630	83
D4	−0.166	0.472	−0.083	5.000	0.394	84
D5	0.193	0.371	0.001	4.651	0.318	83
D6	0.549	0.340	−0.096	3.274	0.287	84
D7	0.861	0.288	0.053	3.012	0.274	83
D8	1.151	0.259	0.057	3.012	0.268	84
D9	1.516	0.341	0.079	3.422	0.266	83
D10	2.154	0.332	−0.044	4.905	0.346	84
**Feeling Safer patients with persecutory delusions (n=89)**						
D1	0.554	0.321	−0.255	3.561	0.294	9
D2	1.082	0.289	−0.041	3.000	0.289	9
D3	1.249	0.232	−0.051	3.333	0.263	9
D4	1.413	0.276	−0.098	3.221	0.253	9
D5	1.566	0.201	−0.124	3.668	0.268	9
D6	1.676	0.249	−0.192	4.000	0.264	8
D7	1.823	0.483	−0.317	4.668	0.269	9
D8	1.989	0.233	0.000	4.781	0.292	9
D9	2.354	0.348	0.050	5.000	0.406	9
D10	2.432	0.271	0.062	5.000	0.421	9

D1=lowest 10% (least paranoia), D10=highest 10% (most paranoia). Mean trait=true theta (standardised score) that is, paranoia level. RMSE values<0.3= high precision and 0.30 to 0.50=acceptable precision. Mean bias reflects accuracy (negative scores=underestimate, positive scores=overestimate). Mean SE reflects measurement precision (lower=more precise; threshold<0.3).

CAT, computerised adaptive testing; RMSE, root mean square error.

The Bland-Altman plot in online supplementalmaterial figure S3 shows a slight negative bias (−0.012), with a mean difference just below zero, indicating that the simulation tended to, on average, slightly underestimate paranoia levels. The majority of points fall within the 95% limits of agreement, which range approximately from −1.0 to +0.5, suggesting that the estimation error was generally within acceptable bounds. A subset of respondents (n=52) (6.22%) fell outside these limits, particularly at the lower end of the trait scale, where estimation appears less precise. Overall, the plot suggests that the simulation provides reasonably accurate theta estimates across most of the paranoia continuum, with only mild systematic bias and a small proportion of outliers.

Based on 50 simulation runs, the subset of individuals with true theta values below 0.4 yielded an RMSE of 0.386 and a mean bias of −0.003 based on the estimated theta values. The RMSE falls within the acceptable precision level. The mean bias reflects a negligible systematic estimation. Approximately 6.1% (n=51) of participants with true theta below 0.4 had an estimated theta that mistakenly placed them in the elevated thresholds, suggesting a low false positive rate. The mean true theta among the misclassified individuals was 0.217, corresponding to an expected test score of 4.352.

### Feeling Safer patients with current persecutory delusions (n=89)

On average, the CAT administered 4.022 items per simulee. The adaptive testing procedure yielded a high correlation of r=0.87 between the true and estimated paranoia values, with a RMSE of 0.299 and a mean bias of −0.10, suggesting on average a slight underestimation of the trait. Only the first item reached the maximum exposure of 1, while the lowest exposure was 0.27. The overall item overlap rate across simulees was 0.506, reflecting moderate test content sharing. Table 4 presents conditional CAT performance metrics across the deciles of paranoia in this participant group.

The Bland-Altman plot in online supplemental material figure S4 shows a slight negative bias (−0.1), with a mean difference just below zero, indicating that the simulation tended to, on average, slightly underestimate paranoia levels. The majority of points fall within the 95% limits of agreement, which range approximately from −0.7 to +0.5, suggesting that the estimation error was generally within acceptable bounds. A small subset of respondents (n=4) (4.49%) fell outside these limits, particularly at the upper ends of the trait scale (which will be very high for this clinical group), where estimation appears less precise. Overall, the plot suggests that the simulation provides reasonably accurate theta estimates across most of the paranoia continuum, with only mild systematic bias and a small proportion of outliers.

This group had persecutory delusions. Only one person had a true theta value below 0.4. For this individual, the estimated theta yielded an RMSE of 0.587 and a mean bias of −0.587.

## Discussion

Collection of patient-reported outcomes is clearly occurring in many mental health services for people with severe mental health difficulties.^39 40^ The challenge is ensuring that this process is informative: that the most relevant data about patients are collected, re-assessed frequently and used to inform treatment provision. Unless such data collection is informative for patients, clinicians and services, it is unlikely to lead to change and will fall by the wayside. Informative is likely to mean that the key clinical problem for a patient that requires treatment is assessed well on a dimensional scale that captures their experience. Tailored testing such as CAT may greatly facilitate the uptake of such meaningful measurement-based care, especially for conditions where there is significant heterogeneity in presentations and hence treatment targets. The efficiency of CAT means that it could be possible to cover multiple domains relatively efficiently.^41^ Hence, key clinical problems for a patient would not be overlooked and could be assessed with precision and accuracy. In this study, we have begun to explore this potential by evaluating how CATs can assess a key presenting problem for many people with psychosis: paranoia. In repeated simulations in clinical and non-clinical datasets, a CAT produced excellent estimates of paranoia compared with full test administration. On average, answers to just four questions were sufficient to produce accurate estimates of a person’s level of paranoia. On average, there was an extremely small tendency to underestimate paranoia. For instance, in the general population group, the mean bias was −0.01, meaning that if a person’s true standardised paranoia score was 1.01 (ie, 1.01 SD above the mean) then the CAT estimate would on average be 1.00. Overall, the results indicate the promise of the CAT approach.

There are limitations. First, the results are from simulations and not the actual administration of a CAT to participants. Therefore, it is unknown whether using a computer interface may have significantly affected the results, though this seems unlikely. Second, it is possible that the patient datasets may have missed sociodemographic or clinical subgroups that could have affected the results. However, we note that we have used multiple datasets, including one dataset with a representative sample of the general population, and the results are similar across them all. Third, accuracy is not fully consistent across the paranoia continuum. We looked at the performance of the CAT across the full range of the paranoia continuum, from people who had no paranoia to people who had the severest clinical forms. Largely, the CAT worked well across the continuum. However, typical for CAT^14^ and as seen in our simulation of a CAT for adolescent paranoia,^25^ it was people at the lower ends of the trait who required the most questions and for whom accuracy was the least good. Ideally, people with the least paranoia would need to answer the fewest number of questions. Nonetheless, five R-GPTS questions did produce sufficient accuracy even for those individuals. What this means is that care should be taken if using the paranoia scale’s severity ranges, as differences by a point or two for people near the boundary could lead to misclassification. Such misclassification is arbitrary and would not be expected to be repeated for an individual as a different set of items would be presented. Fourth, the choice of stopping rules will affect the results. Our intent was to improve the efficiency of the assessment of paranoia, in order to contribute to a future CAT that covers multiple psychotic experiences. Therefore, CAT administration was capped at the presentation of five items. The precision of the assessment could have been improved had such a termination rule not been applied. Importantly, we would now be able to strengthen the CAT algorithm using data from these additional large datasets. Finally, the scale relies on self-report, which could introduce response bias; a proportion of people may not report accurately, and genuine threats may be inadvertently rated. However, our view is that self-report is the best means of capturing what an individual is actually thinking. Furthermore, the GPTS has been shown to correlate with interviewer-based assessments,^26 42^ experimental tests of paranoia^42 43^ and participants’ own sense that their fears are exaggerated.^20 26^ In other words, the scale items do predominately capture inaccurate fears.

## Clinical implications

The results of the study show that a CAT could be programmed from the calibration of the item bank and used successfully for the assessment of paranoia. A CAT for paranoia would be considered a medical device and, as such, needs to be built using best practices in data security. It would be valuable to have a co-design process with patients and mental health staff to find the best ways to present the assessment and the results. Further steps are to develop and test CATs for other dimensional assessments of psychotic experiences and also other important outcome domains. In this way, a comprehensive assessment battery for psychosis—presenting relevant and informative questions to each individual—that has the potential for uptake in services can be realised. Studies of feasibility, acceptability and implementation could be conducted. Repeated, accurate assessment can drive improvement in services. Regular review of an individual’s scores can have many benefits. Perhaps most importantly, it can lead to faster change in treatment provision if the current option is not working well. At a service level, it can facilitate a review of provision if outcomes are not reaching expected benchmarks or matching those of other similar services. Collection of such data at scale can also help identify predictors of outcomes and tailor initial treatment offerings. We believe that the implementation of informative measurement-based care is arguably the best way to achieve a step change in outcomes for patients with psychosis attending clinical services.

## Supplementary material

10.1136/bmjment-2025-302099 online supplemental file 1

## Data Availability

Data are available upon reasonable request.
