# Supplementary material for: Computerised adaptive testing across the paranoia continuum
Source: BMJ Ment Health. 2025 Nov 12;28(1):e302099. doi: 10.1136/bmjment-2025-302099 (PMC12612754; doi:10.1136/bmjment-2025-302099)

**Supplementary materials**

**A. Differential item functioning (DIF) analysis for ethnicity**

DIF analysis was conducted separately using three datasets: a large-scale representative dataset (N=10,382) (Freeman & Loe, 2023), a patient survey dataset (N=1,807) (Freeman et al, 2019), and the non-clinical (OASIS) dataset (N=3,755) (Freeman et al, 2017). The use of multiple, diverse samples is intended to strengthen the evidence concerning measurement invariance. Ethnicity was divided into two main categories (White vs all other ethnicities) to ensure sufficient model stability in the DIF analyses. DIF was evaluated using ordinal logistic regression (Choi et al, 2011). For each item, three nested regression models were compared: (a) a baseline model including the latent trait (θ) estimate as a predictor of item response, (b) a uniform DIF model that added ethnicity as an additional predictor, (c) a non-uniform DIF model that further incorporated an interaction between the θ and ethnicity. Two criteria were applied to evaluate measurement invariance: changes in McFadden’s pseudo R² and regression coefficients (β) associated with ethnicity, and the interaction between ethnicity and θ were examined. Items exhibit negligible DIF if the incremental pseudo R² change is below the recommended threshold (ΔR² < .02) and changes in the regression coefficients do not exceed 10% (Choi et al, 2011; Crane et al, 2007).

In all three datasets, the 10 items demonstrated invariance between White and all other ethnicities, with pseudo ΔR² values of less than 0.02 and changes in regression coefficients of less than 10%. It would be helpful in future studies to have sufficient numbers of participants to examine measurement invariance across all higher-level ethnicity categories.

**References**

Choi, S. W., Gibbons, L. E., & Crane, P. K. (2011). lordif: An R package for detecting differential item functioning using iterative hybrid ordinal logistic regression/item response theory and Monte Carlo simulations. Journal of Statistical Software, 39(8), 1–30.

Crane, P. K., Gibbons, L. E., Narasimhalu, K., Lai, J., & Cella, D. (2007). Rapid detection of differential item functioning in assessments of health-related quality of life: The functional assessment of cancer therapy. Quality of Life Research, 16(1), 101–114.

Freeman, D., & Loe, B. S. (2023). Explaining paranoia: cognitive and social processes in the occurrence of extreme mistrust. BMJ mental health, 26(1).

Freeman, D., Sheaves, B., Goodwin, G. M., Yu, L. M., Nickless, A., Harrison, P. J., ... & Espie, C. A. (2017). The effects of improving sleep on mental health (OASIS): a randomised controlled trial with mediation analysis. The Lancet Psychiatry, 4(10), 749-758.

Freeman, D., Taylor, K. M., Molodynski, A., & Waite, F. (2019). Treatable clinical intervention targets for patients with schizophrenia. Schizophrenia Research, 211, 44-50.

**B. Bland-Altman plots**

Figure S1. UK adult representative dataset (N=10,392)


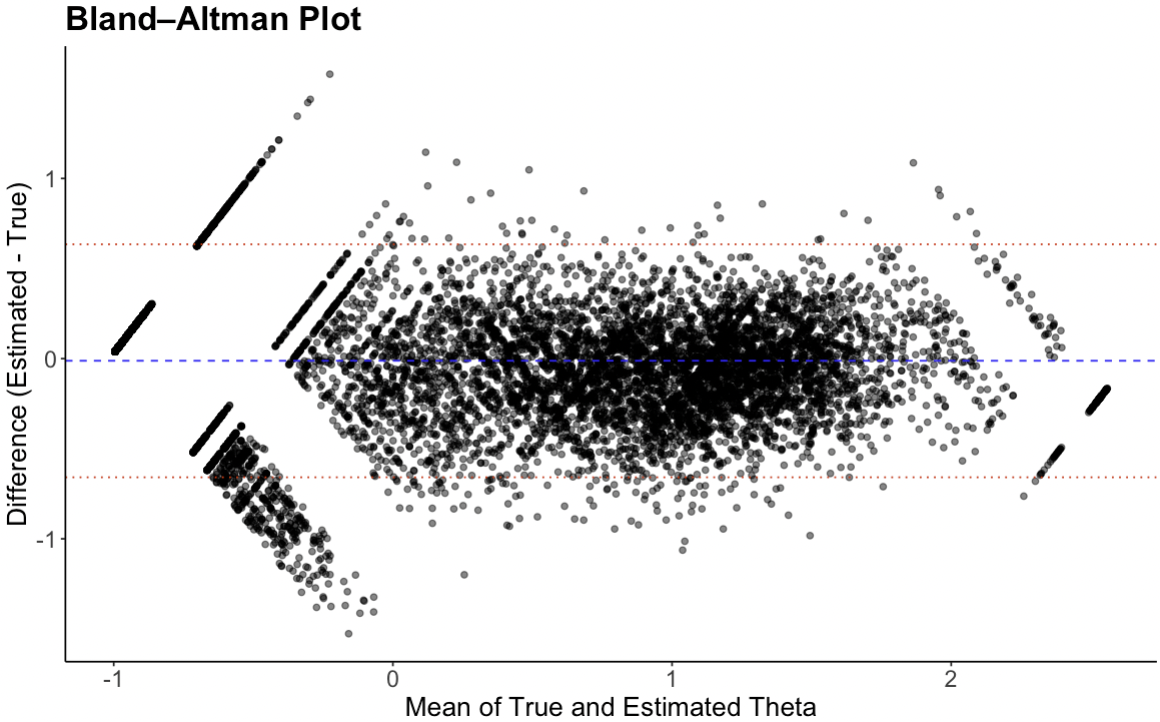


Figure S2. gameChange trial patients with psychosis (N=319).


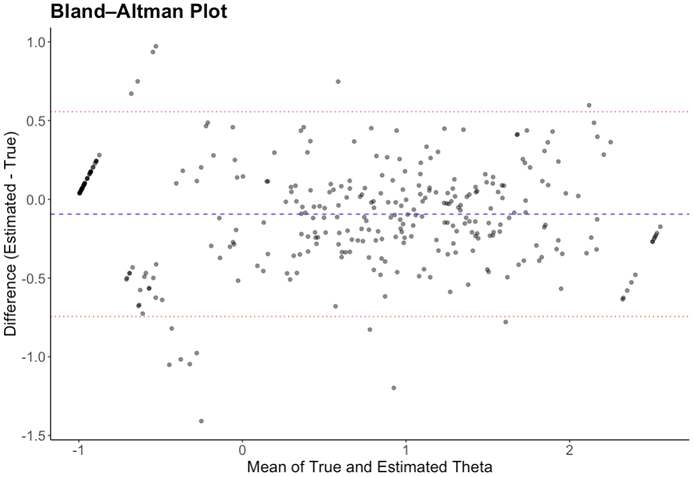


Figure S3. Male patients with psychosis attending NHS mental health trusts (N=836)


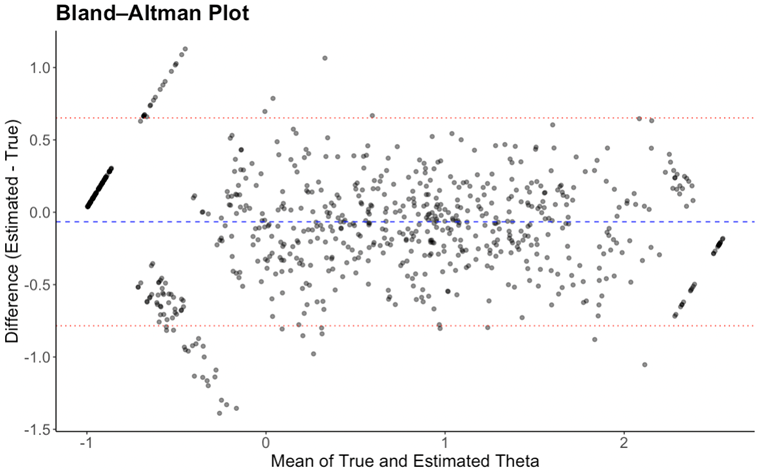


Figure S4. Feeling Safer patients with current persecutory delusions (N=89)


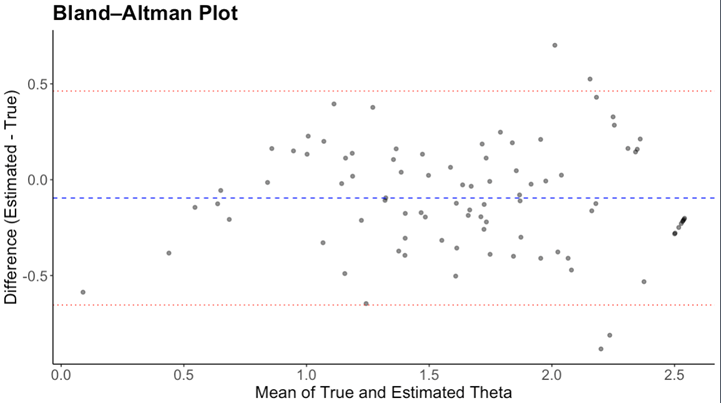

Supplement: online supplemental file 1 [file bmjment-28-1-s001.docx]
